# Dynamic navigation-based precision cardiac rehabilitation via blood metabolomics: from risk stratification to real-time intervention optimization

**DOI:** 10.3389/fcvm.2026.1805494

**Published:** 2026-07-06

**Authors:** Hejuan Hua, Yanbing Chen, Tianyi Yu

**Affiliations:** 1 Department of Cardiology, First People's Hospital of Xiaoshan District, Hangzhou, Zhejiang, China; 2 School of Clinical Medicine, Hangzhou Medical College, Hangzhou, Zhejiang, China; 3 Department of Rehabilitation, First People’s Hospital of Xiaoshan District, Hangzhou, Zhejiang, China

**Keywords:** biomarkers, dynamic navigation, intervention optimization, metabolic fingerprint, metabolomics, precision cardiac rehabilitation, risk stratification

## Abstract

Precision cardiac rehabilitation represents a pivotal approach in the secondary prevention of cardiovascular diseases, yet conventional assessment and intervention models are limited by their static and homogeneous nature. Blood metabolomics, through comprehensive analysis of endogenous small-molecule metabolites, provides a panoramic view of an individual's metabolic status and its dynamic evolution, offering a revolutionary tool for truly personalized and dynamic rehabilitation management. This review introduces the innovative concept of a “dynamic navigation” paradigm, systematically summarizing the application of blood metabolomics in constructing individualized metabolic fingerprints, achieving precise risk stratification, dynamically evaluating rehabilitation responses, and optimizing intervention strategies in real time. We explore the paradigm shift from static biomarkers to dynamic metabolic navigation, addressing the necessary technological integrations and clinical translation challenges inherent in this transition. Furthermore, we discuss future directions toward developing data-driven closed-loop rehabilitation systems that leverage continuous metabolic monitoring to enhance therapeutic efficacy. By bridging metabolomic insights with clinical practice, this dynamic navigation framework promises to transform precision cardiac rehabilitation and improve patient outcomes.

## 1 Introduction

Cardiac rehabilitation (CR) is a comprehensive and long-term management strategy aimed at reducing the risk of recurrent cardiovascular events, enhancing functional status, and improving quality of life for patients with cardiovascular diseases (CVD). Despite its proven benefits, current standardized rehabilitation programs often fail to address the significant heterogeneity among patients. This variability can stem from differences in baseline health status, comorbid conditions, and individual responses to rehabilitation interventions. As a result, there is an urgent need for more personalized approaches in CR that can adapt to the unique profiles of each patient. Recent advancements in blood metabolomics offer a promising avenue for achieving this goal by providing dynamic, real-time insights into the biochemical status of patients, thereby facilitating tailored interventions.

Blood metabolomics, a branch of systems biology, employs high-throughput techniques to quantitatively analyze small-molecule metabolites in biological fluids. These metabolites, which include amino acids, lipids, and organic acids, can reflect the physiological and pathological states of the body as well as the response to therapeutic interventions. By analyzing the “metabolic fingerprint” of an individual, clinicians can gain a deeper understanding of the underlying mechanisms of disease and the potential efficacy of specific rehabilitation strategies. This approach transcends traditional static biomarkers, enabling a more dynamic and responsive method for monitoring patient progress during CR. For instance, studies have shown that metabolomic profiles can be correlated with improvements in cardiac function and exercise capacity, suggesting that targeted interventions based on metabolic data could enhance rehabilitation outcomes (1).

Furthermore, the integration of metabolomics into CR programs allows for the identification of novel biomarkers that can facilitate risk stratification. For example, metabolites related to lipid metabolism, such as triglycerides and phospholipids, have been associated with adverse cardiovascular events. By incorporating these biomarkers into rehabilitation protocols, healthcare providers can better stratify patients according to their risk levels and tailor interventions accordingly. This could lead to more effective management strategies that address the specific needs of high-risk patients, ultimately improving their outcomes and quality of life (2).

However, the application of metabolomics in CR is not without challenges. The complexity of metabolic pathways and the need for sophisticated analytical techniques can pose significant hurdles in translating research findings into clinical practice. Additionally, the variability in individual metabolic responses necessitates a robust understanding of how various factors, such as diet, exercise, and pharmacotherapy, interact with metabolic processes. Addressing these challenges will require collaborative efforts across disciplines, including cardiology, nutrition, and metabolic research, to develop standardized protocols and guidelines for the incorporation of metabolomic data into CR (3).

In conclusion, the integration of blood metabolomics into cardiac rehabilitation represents a transformative opportunity to enhance patient care through precision medicine. By leveraging the dynamic insights provided by metabolic profiling, healthcare providers can develop more individualized rehabilitation strategies that not only improve clinical outcomes but also foster a deeper understanding of the complex interplay between metabolic health and cardiovascular disease. This shift towards a more personalized approach in CR has the potential to redefine the standard of care for patients with cardiovascular conditions, paving the way for improved health outcomes and quality of life.

Dynamic navigation-based precision cardiac rehabilitation workflow driven by blood metabolomics. This closed-loop framework consists of three core sequential stages, which correspond to the content of Section 2 (Baseline Assessment and Risk Stratification), Section 3 (Dynamic Assessment and Real-Time Optimization), and Section 6 (Intelligent Closed-loop Ecosystem) in the main text, respectively.

## 2 Paradigm shift: From static biomarkers to dynamic metabolic fingerprinting navigation

Traditional cardiac rehabilitation relies on discrete, population-based biomarkers and intermittent assessments, which fail to capture real-time metabolic fluctuations or individual heterogeneity. The shift from static single markers to dynamic metabolic fingerprint navigation represents a fundamental change toward truly personalized cardiac rehabilitation. This section clarifies the limitations of conventional tools, defines the metabolic fingerprint concept, and establishes the theoretical framework of the dynamic navigation paradigm.

### 2.1 Limitations of traditional cardiac rehabilitation assessment tools

Traditional cardiac rehabilitation assessment tools, such as cardiopulmonary exercise testing (CPET), echocardiography, and conventional serum biomarkers (e.g., BNP, hs-CRP, troponins), have significant limitations that hinder their effectiveness in capturing the dynamic physiological and metabolic changes that occur during rehabilitation. These tools primarily provide intermittent snapshots of a patient's cardiovascular status, failing to account for the continuous fluctuations in metabolic parameters that can occur in response to rehabilitation interventions. For instance, while CPET can quantify peak oxygen uptake (VO_2_peak), it cannot provide real-time insights into substrate oxidation efficiency or mitochondrial function during daily activity (4). Similarly, conventional inflammatory markers such as high-sensitivity C-reactive protein (hs-CRP) reflect global inflammation but not the specific metabolic pathways driving the inflammatory response (5). Moreover, traditional biomarkers often reflect isolated pathophysiological processes, such as myocardial stretch or inflammation, rather than providing a comprehensive view of the complex, interconnected metabolic networks that influence recovery outcomes. This limitation is particularly critical as it can obscure the understanding of individual patient responses to rehabilitation, leading to a one-size-fits-all approach that may not be effective for all patients (4, 5).

Furthermore, the reliance on population averages for risk stratification and exercise prescription can overlook the significant individual variability in metabolic phenotypes, drug metabolism, and nutritional status among patients. Such an approach may result in inadequate identification of patients at risk for poor rehabilitation outcomes or adverse events, ultimately compromising the effectiveness of the rehabilitation program. Patients with identical clinical profiles may exhibit divergent metabolic responses to the same exercise intensity, leading to suboptimal training outcomes or even cardiovascular stress (6). This lack of personalization can hinder recovery and increase the risk of adverse cardiovascular events during or after rehabilitation (6, 7). Consequently, there is a pressing need for more dynamic and integrative assessment tools that can provide continuous monitoring of physiological and metabolic changes, allowing for real-time adjustments to rehabilitation strategies.

In summary, while traditional assessment tools have been instrumental in the field of cardiac rehabilitation, their limitations in capturing the dynamic nature of patient responses and their reliance on population averages necessitate a shift towards more personalized and continuous assessment methodologies. The transition from baseline risk assessment to longitudinal monitoring is supported by specific metabolic signatures, as summarized in Table 1. These metabolites bridge the gap between initial stratification and adaptive intervention adjustment (15, 16).

**Table 1:** Core blood metabolites and their physiological significance in risk stratification and dynamic monitoring of cardiac rehabilitation.

Rehabilitation stage	Metabolic category/pathway	Representative metabolites	Physiological significance & clinical value
Baseline risk stratification	Branched-chain amino acids (BCAAs) (8)	Leucine, Isoleucine, Valine	Linked to insulin resistance and increased heart failure risk
	Lipids & phospholipids (9)	Sphingolipids, Oxidized phospholipids	Identify high-risk patients with normal routine lipid profiles
	Mitochondrial fatty acid β-oxidation (10)	Acylcarnitines, Carnitine	Early indicator of myocardial energy metabolism dysfunction
Dynamic evaluation (4–12 weeks)	TCA cycle (11)	Citrate, Succinate, Fumarate	Reflect improved mitochondrial function, exercise tolerance, and lower cardiovascular mortality
	Lipid oxidation & ketone metabolism (12)	Acetylcarnitine, β-hydroxybutyrate	Markers of effective aerobic exercise adaptation and energy efficiency
	Inflammation & oxidative stress (13)	Kynurenine, Glutathione, Taurine	Indicate improved endothelial function and plaque stability
	Exercise fatigue & recovery (14)	Lactate, Pyruvate, Purine metabolites	Reflect exercise tolerance and recovery capacity; prevent overtraining

**Figure 1:**
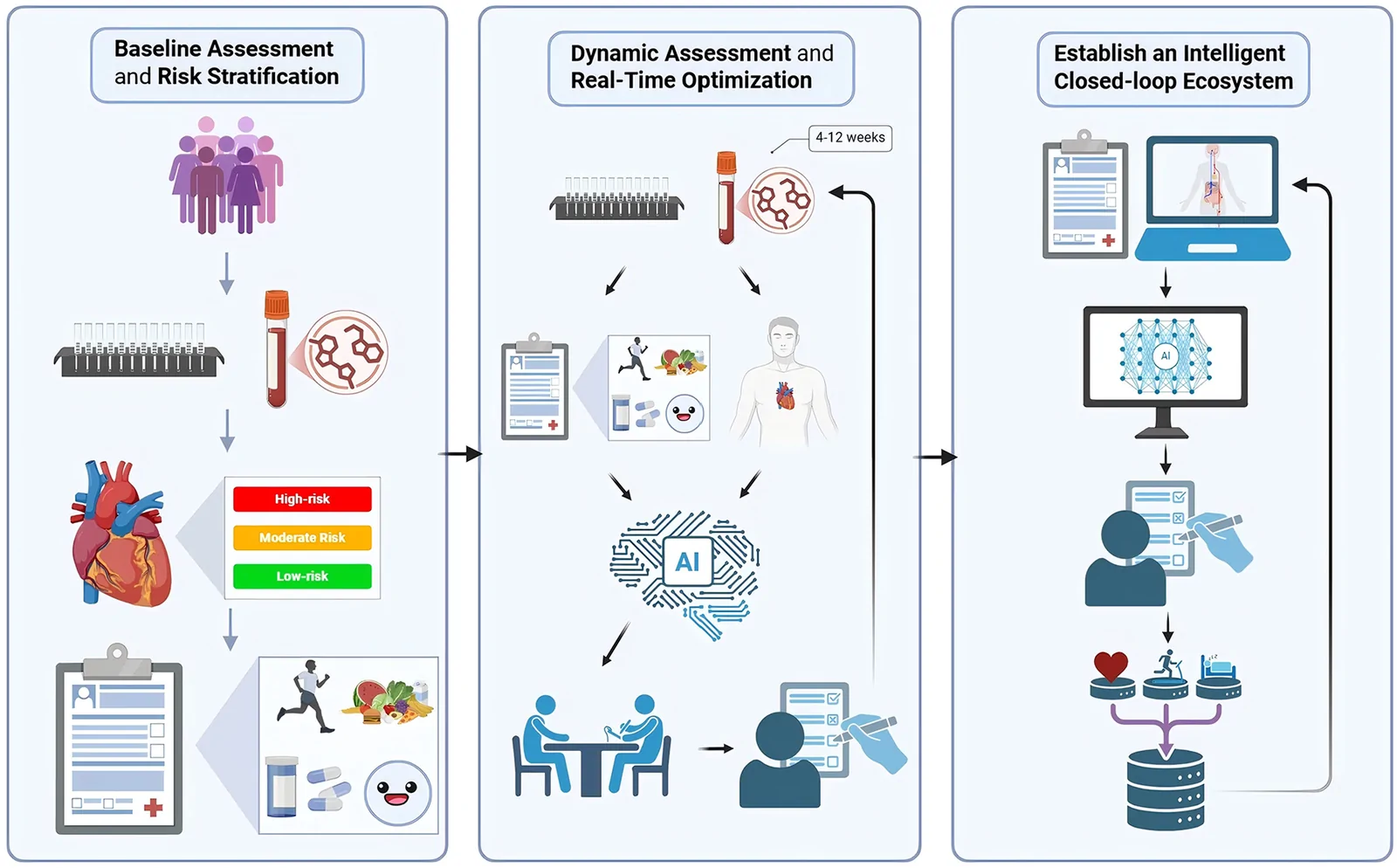
Real-Time intervention optimization flow chart.

In summary, while traditional assessment tools have been instrumental in the field of cardiac rehabilitation, their limitations in capturing the dynamic nature of patient responses and their reliance on population averages necessitate a shift towards more personalized and continuous assessment methodologies. The transition from baseline risk assessment to longitudinal monitoring is supported by specific metabolic signatures, as summarized in Table 1. These metabolites bridge the gap between initial stratification and adaptive intervention adjustment, forming a complete dynamic navigation process together with the workflow shown in Figure 1.

### 2.2 Introduction of metabolomics and the concept of “metabolic fingerprint”

Metabolomics, as a comprehensive approach to small-molecule analysis, offers a unique window into the underlying biochemical state of an individual. By quantifying metabolites in blood or urine, it reflects the integrated outcomes of genetic, environmental, and disease interactions (17). The collective profile of these metabolites constitutes an individual's metabolic fingerprint—a dynamic signature that evolves in response to exercise, nutrition, and pharmacological interventions (18).

This fingerprint serves as a sensitive and specific biomarker. For example, changes in the levels of tricarboxylic acid (TCA) cycle intermediates directly reflect mitochondrial efficiency, while shifts in branched-chain amino acids (BCAAs) indicate metabolic flexibility (19, 20). Unlike static biomarkers, the metabolic fingerprint provides a real-time, holistic view of the body's adaptive response to rehabilitation.

### 2.3 The core connotation and theoretical framework of the “dynamic navigation” paradigm

The “Dynamic Navigation” paradigm represents a transformative approach in cardiac rehabilitation, focusing on the continuous integration of periodically acquired individual metabolic fingerprint data as a central input. This innovative framework employs bioinformatics analyses to perpetually assess a patient's metabolic status, rehabilitation response, and residual risks. By leveraging real-time data, healthcare providers can dynamically optimize a comprehensive intervention plan that encompasses exercise, nutrition, pharmacotherapy, and psychological support. This paradigm shifts the traditional model of cardiac rehabilitation, which often relies on fixed protocols, towards a more personalized and adaptive process that is guided by the patient's ongoing biological feedback. The essence of this approach lies in its ability to tailor interventions to the unique metabolic profiles and evolving needs of each patient, thereby enhancing the effectiveness of rehabilitation strategies and improving patient outcomes (21).

At the heart of the “Dynamic Navigation” paradigm is a closed-loop feedback system that encompasses a cycle of assessment, intervention, and re-assessment. This cyclical process allows for the continuous adjustment of rehabilitation strategies based on real-time data, ensuring that interventions remain relevant and effective as the patient's condition evolves. By moving away from a one-size-fits-all methodology, this paradigm acknowledges the complexity and dynamism inherent in cardiovascular diseases and their rehabilitation. It recognizes that patient responses to interventions can vary widely due to a multitude of factors, including genetic predispositions, lifestyle choices, and psychological states. Thus, the framework emphasizes the importance of personalized medicine, where interventions are not only based on clinical guidelines but are also informed by individual patient data, leading to more nuanced and effective rehabilitation programs (21).

**Figure 2:**
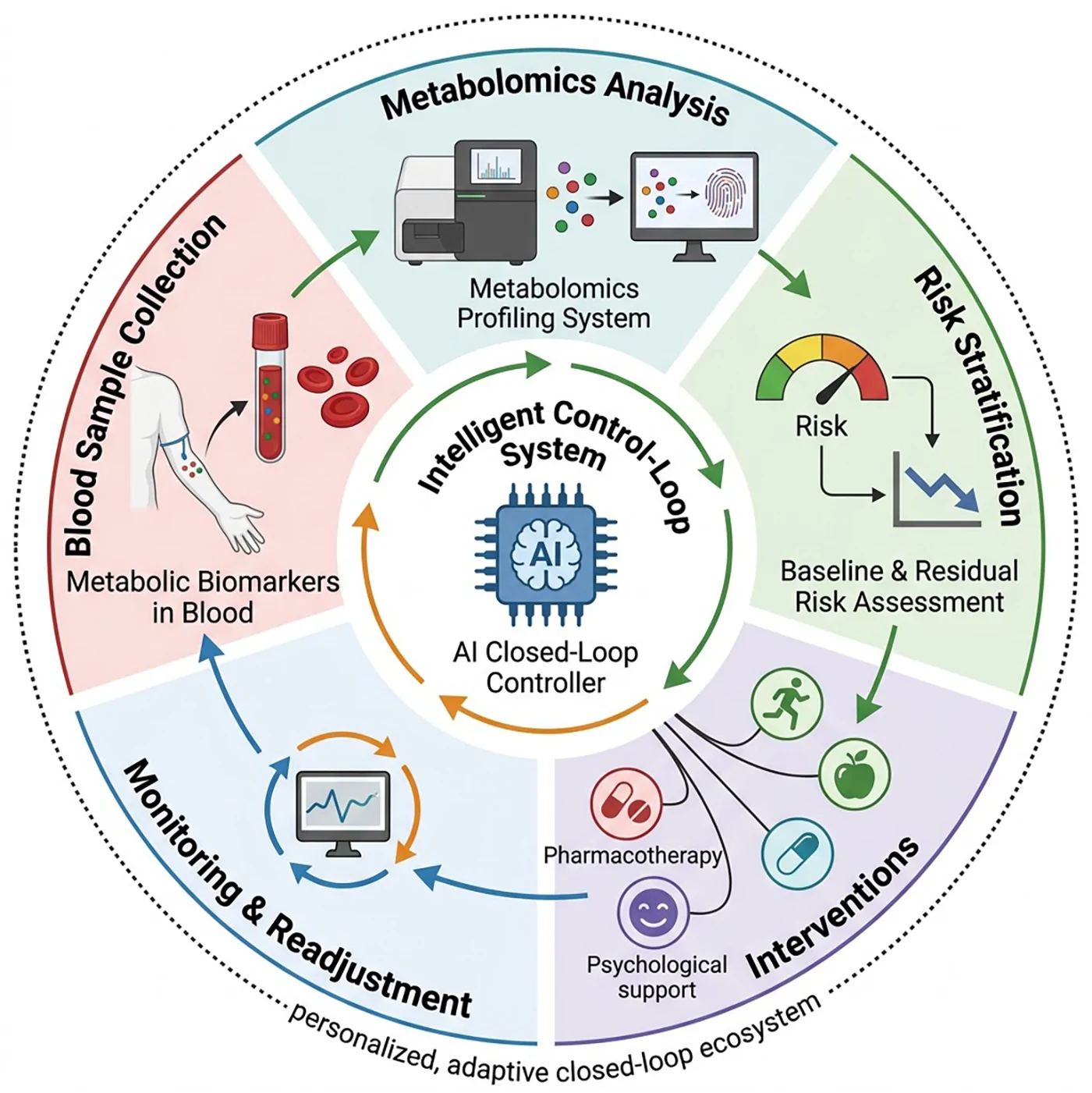
Detailed workflow of the dynamic navigation–guided precision cardiac rehabilitation system.

The theoretical foundation of the “Dynamic Navigation” paradigm is grounded in the recognition that cardiovascular disease and its rehabilitation represent a complex, dynamic systemic issue (Figure 2). This complexity necessitates the use of equally sophisticated and dynamic systemic tools for monitoring and management. The integration of metabolomics and other omics technologies into clinical practice exemplifies this need, as these tools provide insights into the biochemical processes underlying disease states and recovery. By employing such advanced methodologies, healthcare providers can gain a deeper understanding of the metabolic alterations associated with cardiovascular conditions, enabling them to devise targeted interventions that address the root causes of disease rather than merely alleviating symptoms. This systemic approach not only enhances the precision of cardiac rehabilitation but also contributes to the broader goal of improving cardiovascular health outcomes on a population level (22).

The closed-loop framework consists of five sequential and iterative phases: (1) blood sample collection and metabolomic profiling to generate individual metabolic fingerprints; (2) baseline risk stratification and residual risk evaluation using core metabolic biomarkers; (3) dynamic assessment of rehabilitation response during 4–12 weeks of intervention; (4) real-time optimization of exercise, nutrition, pharmacotherapy, and psychological support; and (5) continuous monitoring and readjustment to form an intelligent closed-loop ecosystem. This workflow enables personalized and adaptive cardiac rehabilitation tailored to individual metabolic status.

Growing evidence from clinical cardiac rehabilitation cohorts has validated that real-time metabolomic monitoring can guide adaptive intervention adjustments and improve exercise tolerance and clinical outcomes compared with conventional static protocols (23). A multicenter prospective study further demonstrated that metabolic fingerprint–guided dynamic navigation reduced the incidence of major adverse cardiovascular events (MACE) and shortened rehabilitation cycles in post–myocardial infarction patients (8). These clinical data provide solid cohort-based support for the feasibility and effectiveness of the dynamic navigation paradigm.

## 3 Application of metabolic fingerprinting in risk stratification and prognostic prediction in cardiac rehabilitation

Metabolic fingerprints enable precise risk stratification at baseline and serve as powerful predictors of long-term outcomes. This section elaborates on how baseline metabolites identify residual risks and how early dynamic changes forecast recovery, integrating the latest clinical evidence and mechanistic insights into cardiovascular rehabilitation practice.

### 3.1 Baseline metabolic fingerprints and identification of residual cardiovascular risk

Recent studies have demonstrated that specific baseline metabolic profiles are significantly correlated with adverse cardiovascular outcomes, thereby serving as potential biomarkers for cardiovascular risk stratification. A key example is the elevation of branched-chain amino acids (BCAAs)—including leucine, isoleucine, and valine—which has been closely linked to insulin resistance and an increased risk of heart failure (9). For instance, a preliminary study on myocardial infarction patients undergoing CR found that plasma BCAA levels correlate with disease severity, further supporting their utility as baseline risk markers (10).

Furthermore, lipidomic analyses have revealed that specific phospholipid and sphingolipid species, such as phosphatidylcholine (PC) and ceramides, provide incremental risk information beyond conventional lipid profiles (11). These metabolites can identify high-risk individuals who may present with normal traditional lipid markers but exhibit metabolic abnormalities that elevate their cardiovascular risk (12).

In conclusion, the integration of baseline metabolic fingerprints into cardiovascular risk assessment represents a promising approach to identifying residual risk in patients undergoing CR. By leveraging advances in metabolomics, clinicians can gain deeper insights into the biochemical underpinnings of cardiovascular diseases, facilitating the development of targeted interventions that address the unique metabolic profiles of individual patients. The ongoing research in this field, including studies utilizing minimally invasive dried blood spot (DBS) sampling for metabolic profiling, holds the potential to significantly improve cardiovascular health outcomes through more precise risk stratification and personalized treatment strategies.

### 3.2 Dynamic changes in metabolic fingerprints during early rehabilitation and prediction of long-term prognosis

The initiation of rehabilitation programs, particularly within the timeframe of 4–12 weeks post-intervention, has been shown to yield significant alterations in metabolic fingerprints that are more predictive of long-term outcomes than baseline measurements (Table 2). For instance, Positive changes in TCA cycle intermediates including citrate and succinate are associated with enhanced mitochondrial function, better exercise tolerance, and reduced cardiovascular mortality (24). This enhancement in mitochondrial activity is linked to better exercise tolerance and a reduction in cardiovascular mortality rates. The ability to monitor these metabolic shifts provides clinicians with a valuable tool for assessing patient progress and tailoring rehabilitation strategies accordingly. Moreover, early metabolic adaptations can serve as biomarkers for recovery, offering insights into the physiological changes that accompany rehabilitation efforts (25).

**Table 2:** Summary of clinical studies on metabolomics in cardiac rehabilitation cohorts.

Study	Year	Population	Target metabolites	Key findings	Clinical outcome
Ghorbani et al.	2024	Patients undergoing exercise-based CR	Acylcarnitines, BCAAs, TCA cycle intermediates	Metabolic profiles dynamically reflect rehabilitation response; early changes predict functional improvement	Improved exercise tolerance and quality of life
van der Meer et al.	2025	Post-myocardial infarction patients	Lipids, oxidized phospholipids, kynurenine	Metabolic fingerprint–guided intervention reduces MACE risk	Lower MACE incidence, shorter rehabilitation duration
Cheng et al.	2017	Heart failure patients	BCAAs, sphingolipids	Metabolic markers improve risk stratification beyond traditional biomarkers	Enhanced prognostic accuracy
Wang et al.	2023	Coronary artery disease patients	Lactate, arhydroxybutyrate	Metabolic markers quantify exercise response and identify non-responders	Optimized exercise prescription
Rehman et al.	2025	Post-PCI CR patients	Carnitine, glutathione	Metabolic normalization correlates with endothelial function improvement	Reduced hospital readmission

In addition to mitochondrial function, the normalization of metabolic pathways associated with inflammation and oxidative stress—such as the tryptophan-kynurenine pathway and glutathione metabolism—can serve as indicators of improved anti-inflammatory and antioxidant status. The tryptophan-kynurenine pathway, the primary route for tryptophan degradation, produces metabolites (e.g., kynurenine) that are elevated in various cardiovascular diseases and mediate inflammation through aromatic hydrocarbon receptor (AHR) activation; its early normalization during CR suggests a beneficial impact on plaque stability and endothelial function, which are critical components of cardiovascular health (12). Patients exhibiting these metabolic changes may experience not only a reduction in inflammatory markers but also an enhancement in vascular health, further supporting the notion that metabolic profiling can provide predictive insights into long-term cardiovascular outcomes.

Notably, the integration of baseline and early dynamic metabolic profiles using machine learning models has shown promise for constructing composite predictors of readmission risk and major adverse cardiovascular events (MACE) (24). Such data-driven models often outperform conventional clinical risk scores in stratification performance. However, this predictive potential remains largely hypothetical in the cardiac rehabilitation population and is primarily supported by evidence derived from other clinical cohorts, including general cardiovascular patients, heart failure cohorts, or post-myocardial infarction populations rather than dedicated cardiac rehabilitation cohorts.

Several important limitations must be acknowledged when extrapolating these findings to patients undergoing structured cardiac rehabilitation. First, most existing machine learning models lack prospective validation in longitudinal rehabilitation settings, where metabolic responses are continuously modified by exercise, nutrition, and pharmacotherapy. Second, metabolic profiles in rehabilitation patients are highly dynamic and may be confounded by changes in physical activity, medication adherence, and comorbidity management, which differ substantially from stable outpatient cohorts. Third, the generalizability of predictive signatures remains limited by heterogeneity in age, baseline function, comorbidities, and intervention intensity across studies. Therefore, while metabolomic-based predictive modeling represents a promising direction, further prospective, multi-center studies specifically designed for cardiac rehabilitation populations are required before clinical implementation (26).

Collectively, early dynamic changes in metabolic fingerprints reflect meaningful physiological adaptations and carry prognostic implications. However, their translation into clinical decision-making for cardiac rehabilitation requires cautious interpretation, rigorous validation, and explicit consideration of population-specific limitations.

## 4 The role of metabolic fingerprinting in dynamic evaluation of rehabilitation effects and intervention feedback

### 4.1 Quantifying metabolic responses to exercise interventions

**Figure 3:**
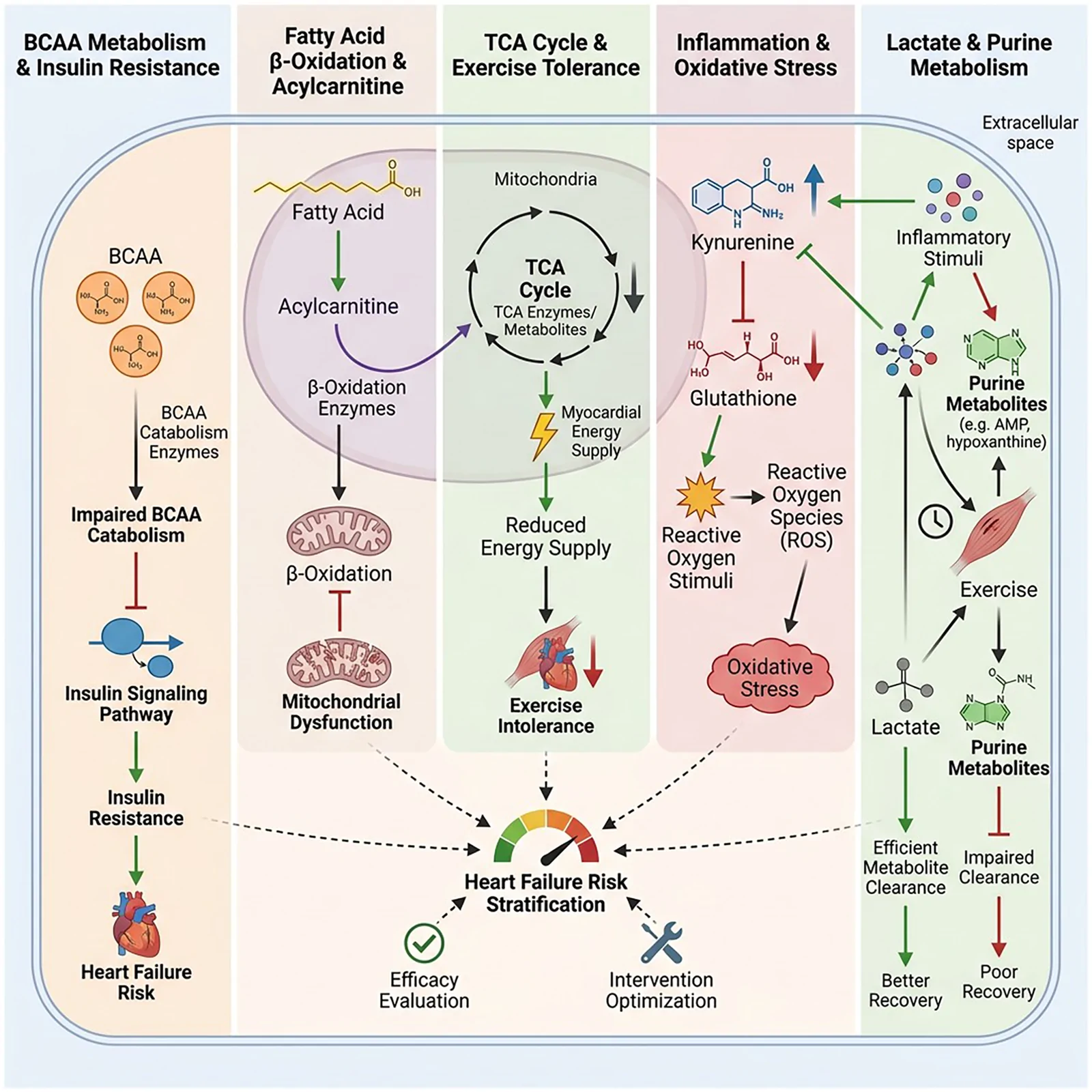
Key metabolic pathways and their clinical relevance in precision cardiac rehabilitation.

The optimization of exercise prescriptions, including intensity, type, and duration, is at the core of effective cardiac rehabilitation (Figure 3). Individualized exercise regimens are essential to ensure that each patient receives the most beneficial training tailored to their unique physiological responses. Metabolomics, the comprehensive study of metabolites in biological samples, offers an objective means to quantify the metabolic adaptations induced by exercise. For instance, aerobic exercise has been shown to increase metabolites associated with fat oxidation, such as acetylcarnitine and β-hydroxybutyrate, which are critical indicators of enhanced lipid metabolism and energy utilization during prolonged exercise sessions (27). In contrast, resistance training appears to have a more pronounced effect on the amino acid metabolic profile, particularly those related to protein synthesis, highlighting the differing metabolic pathways activated by various forms of exercise (27). This differentiation is crucial for tailoring exercise interventions that not only promote cardiovascular health but also optimize metabolic health based on individual patient profiles.

Monitoring the recovery dynamics of specific metabolites post-exercise, such as lactate and purine metabolites, provides valuable insights into an individual's exercise tolerance and recovery capacity. The kinetics of lactate clearance, for example, can inform clinicians about a patient's ability to handle exercise intensity and adjust the training load accordingly (28). Elevated lactate levels post-exercise indicate a higher reliance on anaerobic metabolism, while a rapid return to baseline levels suggests efficient metabolic recovery and a well-conditioned state. Similarly, the assessment of purine metabolites can reveal insights into cellular energy status and recovery processes, allowing for a more nuanced approach to exercise prescription that accommodates the specific needs and capabilities of each patient (28). This real-time monitoring of metabolic responses not only aids in optimizing exercise intensity but also enhances patient safety by preventing overtraining and ensuring adequate recovery.

Identifying specific metabolic characteristics of “non-responders” to exercise interventions is another critical aspect of utilizing metabolomics in cardiac rehabilitation. Metabolic inflexibility is defined as the impaired ability to switch substrate utilization between carbohydrates and fatty acids in response to physiological demands such as fasting, feeding, or exercise (13). The underlying mechanisms mainly involve mitochondrial dysfunction, incomplete fatty acid oxidation, and abnormal BCAA catabolism, leading to insufficient energy supply and increased oxidative stress during rehabilitation (28). At the metabolite level, metabolic inflexibility is characterized by blunted acylcarnitine production, persistently elevated BCAAs, reduced TCA cycle flux, and impaired lactate clearance kinetics (29). Certain individuals may exhibit persistent abnormalities in mitochondrial metabolites, which could indicate underlying biological mechanisms that hinder their response to conventional exercise programs (29). Understanding these unique metabolic profiles allows clinicians to explore alternative intervention. Understanding these unique metabolic profiles allows clinicians to explore alternative interventions, such as nutritional supplementation or pharmacological adjustments, to enhance the effectiveness of rehabilitation strategies tailored for these patients (30). For instance, if a patient is identified as a non-responder due to metabolic inflexibility, interventions such as targeted dietary changes or specific nutrient supplementation could be employed to improve their metabolic health and overall response to exercise (14). This personalized approach not only enhances the efficacy of rehabilitation programs but also fosters a more holistic understanding of the interplay between exercise, metabolism, and individual patient characteristics in the context of cardiac recovery.

Beyond macro-physiological improvements, the distinct impact of various exercise intensities on mitochondrial metabolic flux provides a deeper layer of granularity to rehabilitation monitoring. The metabolic signature of exercise is highly intensity-dependent, reflecting distinct metabolic flux through mitochondrial pathways. High-Intensity Interval Training (HIIT) triggers a robust metabolic challenge characterized by rapid flux in the TCA cycle and significant transient increases in lactate, pyruvate, and acylcarnitines, signaling an acute reliance on anaerobic glycolysis and rapid lipid mobilization. In contrast, Moderate Continuous Training (MCT) promotes a more sustained aerobic adaptation, enhancing the efficiency of lactate clearance kinetics and mitochondrial oxidative phosphorylation. Specifically, the dynamic shift in the succinate-to-fumarate ratio post-HIIT serves as a metabolic sensor for mitochondrial remodeling, while MCT is more associated with the long-term stabilization of lipidomic profiles and amino acid homeostasis.

Five core pathways are highlighted: (1) branched-chain amino acid (BCAA) metabolism linked to insulin resistance and heart failure risk; (2) fatty acid β-oxidation and acylcarnitine metabolism reflecting mitochondrial function; (3) tricarboxylic acid (TCA) cycle associated with exercise tolerance and myocardial energy supply; (4) inflammation and oxidative stress pathways involving kynurenine and glutathione; and (5) lactate and purine metabolism indicating exercise recovery capacity. Alterations in these pathways serve as critical biomarkers for risk stratification, efficacy evaluation, and intervention optimization.

### 4.2 Guiding personalized nutrition and pharmacological interventions

Metabolomics, particularly nutritional metabolomics, serves as a powerful tool for assessing the actual nutritional status of patients, including critical parameters such as vitamin B status and essential fatty acid levels. This approach enables clinicians to tailor dietary interventions based on individual metabolic responses. For instance, the supplementation of Omega-3 fatty acids has been shown to induce changes in specific lipid mediators, such as resolvins and protectins, which are involved in the resolution of inflammation. These metabolites can serve as biomarkers to evaluate the anti-inflammatory effects of dietary interventions, thus providing a clear pathway for personalized nutrition strategies that optimize patient outcomes in cardiac rehabilitation and recovery processes (31). Furthermore, the integration of metabolomic data into clinical practice can enhance the understanding of how individual patients respond to dietary changes, leading to more effective and customized nutritional support.

In the realm of pharmacological management, metabolomics plays a vital role in monitoring the effects of medications, particularly statins, on cholesterol synthesis pathways, specifically the mevalonate pathway. By analyzing metabolites associated with cholesterol metabolism, healthcare providers can assess the efficacy of statin therapy in individual patients. For example, the levels of cholesterol and its precursors can be monitored to evaluate the therapeutic response and adjust treatment plans accordingly. Additionally, metabolomics can be employed to assess the metabolic activation of antiplatelet drugs like clopidogrel, where the levels of its active metabolites can indicate the drug's efficacy and safety profile. This individualized assessment becomes critical in minimizing adverse effects, such as statin-associated myopathy, thereby enhancing patient safety and treatment adherence (31). The ability to tailor pharmacological interventions based on metabolic profiles not only optimizes therapeutic outcomes but also empowers patients in their treatment journeys.

Moreover, metabolomic profiling can reveal significant drug-nutrient interactions that may alter therapeutic efficacy or lead to adverse reactions. For instance, individual metabolic differences can result in the attenuation of the intended effects of dietary supplements or medications, necessitating a personalized approach to treatment. By identifying these interactions through metabolomic analysis, healthcare providers can avoid potential pitfalls associated with standard treatment protocols. This personalized approach is especially crucial in populations with diverse genetic backgrounds, where variations in metabolic pathways can significantly influence drug metabolism and nutrient utilization (32).

## 5 Technical platforms and data analysis strategies for implementing dynamic navigation

### 5.1 Cutting-edge analytical techniques and standardized processes

The evolution of metabolomics has been significantly influenced by advanced analytical techniques, particularly liquid chromatography-mass spectrometry (LC-MS) and gas chromatography-mass spectrometry (GC-MS). These methodologies are at the forefront of metabolomic analysis due to their high throughput, sensitivity, and broad coverage of metabolites. Recent advancements in LC-MS and GC-MS have focused on enhancing the resolution and accuracy of metabolite identification and quantification, enabling researchers to analyze complex biological samples with unprecedented detail. For instance, the integration of high-resolution mass spectrometry (HRMS) with these chromatographic techniques has allowed for the identification of hundreds of metabolites in a single run, facilitating comprehensive metabolic profiling (33). Additionally, the development of standardized protocols for sample preparation and data analysis is critical for ensuring reproducibility and comparability across studies. This standardization is essential for the establishment of metabolomic databases that can be utilized for cross-species analyses and meta-analyses, thereby enhancing the field's overall robustness and reliability (34).

The advent of micro-sampling techniques, such as dried blood spots (DBS), and point-of-care testing (POCT) devices represents a pivotal shift towards more frequent and convenient metabolic monitoring. These innovations are particularly crucial for dynamic navigation in precision cardiac rehabilitation, as they allow for real-time monitoring of metabolic changes in response to interventions. DBS, for instance, enables the collection of small blood volumes, which can be easily transported and analyzed without the need for extensive laboratory infrastructure. This method not only simplifies the logistics of sample collection but also minimizes the risk of contamination and degradation of metabolites during transport (35). Furthermore, the development of POCT devices that can perform rapid metabolic analyses at the bedside has the potential to revolutionize patient care by providing immediate feedback on metabolic status, thus facilitating timely adjustments to treatment plans (36). These technological advancements are essential for the implementation of a dynamic, real-time approach to metabolic monitoring in clinical settings.

Establishing a comprehensive and standardized workflow from sample collection to data interpretation is foundational for the advancement of metabolomics in clinical applications. This includes the development of protocols that address every step of the metabolomics workflow, such as sample collection, preprocessing, data acquisition, and analysis. Quality control measures must be integrated at each stage to ensure the reliability and reproducibility of data. For instance, the use of reference standards and internal controls during mass spectrometry analysis can help mitigate variations caused by instrument performance and sample handling (37). Furthermore, adopting standardized reporting guidelines will enhance the transparency and comparability of metabolomic studies, allowing for better integration of findings into clinical practice (38). By establishing robust quality assurance and quality control systems, researchers can ensure that metabolic data are not only comparable across studies but also translatable into meaningful clinical insights, ultimately supporting the goal of personalized medicine in cardiac rehabilitation and beyond.

### 5.2 Integration of multi-omics data and AI-driven models

The integration of multi-omics data, including metabolomics, genomics, proteomics, and microbiomics, represents a significant advancement in our understanding of complex diseases and the development of personalized medicine. By combining these diverse data types, researchers can create comprehensive biological profiles of patients, leading to a deeper understanding of disease mechanisms and responses to interventions. For instance, integrating metabolomics data with genomic information related to drug metabolism can help identify genetic polymorphisms that influence individual responses to treatments. This integration enhances our ability to stratify patients based on their unique biological characteristics, allowing for more tailored therapeutic approaches. Furthermore, the inclusion of microbiome data, particularly metabolites produced by gut microbiota, can provide insights into the interactions between diet, metabolism, and disease, thereby refining our understanding of health and disease dynamics (39, 40).

Artificial intelligence (AI) and machine learning algorithms play a crucial role in processing the high-dimensional and complex datasets generated from multi-omics studies. These computational tools, such as random forests and deep learning models, are adept at identifying key metabolic features and constructing predictive models that can inform clinical decision-making. For example, machine learning techniques can be employed to analyze large-scale omics datasets, enabling the identification of biomarkers that predict disease outcomes or treatment responses. The ability of AI to discern patterns in complex datasets facilitates the development of decision support systems that can assist clinicians in making informed choices about patient care. This is particularly relevant in fields like oncology and cardiology, where the integration of multi-omics data can lead to significant improvements in diagnostic accuracy and treatment personalization (41, 42).

To bridge the gap between laboratory findings and clinical application, it is essential to develop user-friendly clinical decision support interfaces that translate complex multi-omics data into actionable clinical recommendations. Such interfaces can empower healthcare providers by presenting intuitive visualizations of patient data, highlighting critical biomarkers, and suggesting tailored interventions based on individual patient profiles. The integration of AI-driven analytics into these platforms can enhance their functionality, enabling real-time monitoring and adaptive treatment strategies that evolve as new data becomes available. By facilitating the seamless transition from research to bedside, these technological advancements hold the potential to revolutionize patient care and improve health outcomes across various medical disciplines (43, 44).

Overall, the integration of multi-omics data with AI-driven models represents a transformative approach in precision medicine. By leveraging the strengths of diverse biological datasets and advanced computational techniques, researchers and clinicians can gain unprecedented insights into disease mechanisms, optimize therapeutic interventions, and enhance patient care. As these technologies continue to evolve, they promise to unlock new avenues for understanding health and disease, ultimately leading to more effective and personalized healthcare solutions (45, 46).

### 5.3 Addressing the “non-responder” phenotype through metabolic rescue

A pivotal challenge in achieving universal rehabilitation success lies in the biological heterogeneity of patients, particularly those categorized as “non-responders” who fail to achieve expected functional gains.

A critical hurdle in standard CR is the existence of “non-responders”—individuals who show minimal improvement in functional capacity despite adherence to exercise protocols. Metabolomics offers a window for early identification; non-responders often exhibit a blunted acylcarnitine response and persistent elevations in branched-chain amino acids (BCAAs), suggesting impaired mitochondrial flexibility (47). For these individuals, the “dynamic navigation” system should trigger a “Rescue Protocol”: integrating pharmacological support (e.g., trimetazidine to optimize glucose oxidation) or targeted nutritional supplementation (e.g., Coenzyme Q10 or L-carnitine) to bypass metabolic bottlenecks and facilitate functional recovery.

## 6 Challenges and strategies in clinical translation

### 6.1 Scientific validation and clinical efficacy evidence

Current metabolic research in the realm of cardiac rehabilitation is predominantly in the discovery and validation phases, lacking the robust backing of large-scale, prospective, multi-center clinical trials that can substantiate the superiority of dynamic navigation models over standard care protocols, particularly in terms of improving hard endpoints. The promise of metabolomics lies in its potential to provide real-time insights into the biochemical changes occurring in patients undergoing rehabilitation, which could facilitate personalized interventions tailored to individual metabolic profiles. However, the absence of comprehensive studies that compare these innovative approaches directly with conventional rehabilitation methods limits the ability to draw definitive conclusions about their clinical effectiveness. For example, while studies have shown that specific metabolites can correlate with improved cardiac function post-myocardial infarction, as seen with the elevation of isatin levels in STEMI patients undergoing Enhanced External Counterpulsation (EECP) rehabilitation, the lack of extensive clinical trials means that the generalizability of these findings remains uncertain (48). Moreover, the identification of such biomarkers must be accompanied by rigorous validation processes to ensure their reliability and applicability in diverse clinical settings.

To further advance the field, it is imperative to establish clear, clinically relevant thresholds for metabolite changes that signify meaningful clinical implications. This involves determining the extent of metabolic alterations that can be considered significant enough to warrant changes in therapeutic strategies. For instance, the development of a metabolomics detection panel specifically designed for distinct rehabilitation scenarios—such as heart failure recovery or post-percutaneous coronary intervention (PCI) rehabilitation—could provide clinicians with actionable insights that enhance patient outcomes. The creation of such panels would require meticulous validation against established clinical outcomes to ensure that the identified metabolite changes are not only statistically significant but also translate into tangible improvements in patient health. The findings from studies investigating the effects of Hydroxyl Safflower Yellow A (HSYA) on myocardial ischemia-reperfusion injury illustrate the potential of targeted metabolomic interventions. HSYA was shown to activate the HIF-1α-VEGFA-Notch1 signaling pathway, promoting angiogenesis and thereby enhancing cardiac function (49). This highlights the need for a more profound understanding of the underlying mechanisms through which metabolic changes impact rehabilitation outcomes, as well as the necessity for clinical trials that can validate these mechanisms in a real-world context.

### 6.2 Cost-effectiveness, accessibility, and ethical considerations

The integration of metabolomics into dynamic navigation-based cardiac rehabilitation presents significant cost-effectiveness challenges that must be addressed through comprehensive health economic evaluations. Currently, the high costs associated with metabolomic analyses can be a barrier to widespread implementation in clinical practice. To justify the investment in such advanced technologies, it is essential to demonstrate that precision interventions based on metabolomic data can lead to substantial long-term savings by reducing complications and readmissions. For instance, studies have shown that personalized rehabilitation strategies can significantly improve patient outcomes, potentially decreasing the need for costly hospitalizations due to heart failure or other cardiac events (50). A detailed economic assessment could provide insights into the cost-benefit ratio of implementing metabolomics in cardiac rehabilitation, ensuring that the financial implications are favorable when considering the overall healthcare expenditure. Moreover, as healthcare systems increasingly adopt value-based care models, demonstrating the financial viability of metabolomics will be crucial for securing funding and support from stakeholders, including healthcare providers and policymakers.

Ensuring equitable access to metabolomics-driven cardiac rehabilitation is another critical consideration. The implementation of such advanced technologies must not exacerbate existing disparities in healthcare access, particularly among underserved populations. It is vital to develop strategies that promote fair distribution of resources and ensure that all patients, regardless of socioeconomic status, can benefit from these innovations. This includes addressing potential barriers such as geographical limitations, healthcare literacy, and financial constraints that may prevent certain groups from accessing metabolomic assessments and tailored rehabilitation programs. Furthermore, ethical considerations surrounding data privacy and informed consent must be prioritized. Patients should be adequately informed about how their metabolic data will be used and the implications of such analyses on their treatment plans. Clear communication strategies must be developed to help both patients and healthcare providers understand the complexities of metabolomic data and its relevance to individualized care (51).

The successful implementation of dynamic navigation-based rehabilitation programs also hinges on the development of interdisciplinary teams equipped with the necessary skills in metabolomics, data science, and clinical practice. Building a workforce that is proficient in these areas will be essential for the effective integration of metabolomics into routine cardiac rehabilitation. Training programs should focus on enhancing the knowledge and competencies of healthcare professionals, enabling them to interpret complex metabolic data and apply it to patient care effectively. This collaborative approach will not only improve the quality of care provided to patients but also foster an environment of innovation and continuous learning within healthcare organizations. By cultivating a diverse team of experts, healthcare facilities can ensure that they are well-prepared to navigate the challenges associated with implementing advanced technologies in cardiac rehabilitation, ultimately leading to improved patient outcomes and satisfaction (52).

### 6.3 Limitations and speculative nature of the dynamic navigation concept

Despite promising preliminary findings, the dynamic navigation paradigm based on blood metabolomics still has inherent limitations and speculative components. First, most existing evidence is derived from small-sample, single-center observational studies; large-scale randomized controlled trials (RCTs) with hard clinical endpoints are lacking (53). Second, the cutoff values of key metabolites for clinical decision-making remain unstandardized, and real-time point-of-care metabolomic testing is not widely available in routine clinical settings (54). Third, the long-term safety and cost-effectiveness of continuous metabolic monitoring during cardiac rehabilitation have not been fully verified (55). Therefore, the dynamic navigation framework is still at the exploratory stage, and its clinical translation requires further rigorous validation rather than being regarded as a mature clinical standard.

## 7 Future outlook: building an intelligent closed-loop cardiac rehabilitation ecosystem

### 7.1 Integration of wearable devices and digital therapeutics

The future of personalized cardiac rehabilitation lies in the seamless integration of continuous monitoring data from wearable devices with intermittent metabolic fingerprint data, facilitated by digital twin technology. Wearable devices, such as smartwatches and fitness trackers, are capable of capturing a plethora of physiological parameters including heart rate variability, physical activity levels, and sleep patterns. This continuous stream of real-time data can be harnessed to create a dynamic and comprehensive profile of a patient's health status. By combining this data with metabolic information obtained through periodic assessments—such as blood metabolomics or breath analysis—healthcare providers can construct a virtual metabolic-physiological model of the patient. This model serves as a digital twin, allowing for a more nuanced understanding of the individual's health and response to interventions. The application of digital twin technology in this context not only enhances the accuracy of risk stratification but also facilitates tailored interventions that adapt to the patient's evolving health status over time. This integration is pivotal for optimizing cardiac rehabilitation strategies, as it allows for the identification of specific health trends and potential risks that may require immediate attention.

Building on the insights gained from the digital twin model, digital therapeutic applications can provide real-time, personalized recommendations for exercise, nutrition, and medication adherence. These applications can analyze the data from wearable devices and metabolic assessments to deliver customized guidance that aligns with the patient's unique health profile and rehabilitation goals. For instance, if a patient's wearable device indicates a decline in physical activity or an increase in heart rate variability, the digital therapeutic application can alert both the patient and their healthcare provider, prompting timely interventions. This creates a closed-loop system of “monitoring-analysis-intervention-re-monitoring,” where each phase informs the next, ensuring that the rehabilitation process is continuously optimized based on the patient's current health status. Such a system not only enhances patient engagement and adherence to rehabilitation protocols but also empowers healthcare providers to make data-driven decisions that can significantly improve patient outcomes.

### 7.2 Extension of the paradigm from disease management to health promotion

The concept of dynamic metabolic navigation extends beyond the management of cardiovascular diseases to encompass primary prevention and health management in healthy populations. This approach emphasizes the early identification of subclinical metabolic disorders, allowing for proactive lifestyle interventions that can significantly alter disease trajectories. By leveraging metabolic profiling, healthcare providers can identify individuals at risk for cardiovascular diseases even before clinical symptoms manifest. This proactive strategy facilitates tailored interventions that promote healthier lifestyle choices, such as dietary modifications and increased physical activity, which are crucial for preventing the onset of cardiovascular conditions. Moreover, the integration of metabolic data into routine health assessments can empower individuals to take charge of their health, fostering a culture of prevention rather than reaction. This shift in focus from disease management to health promotion aligns with the growing recognition of the importance of lifestyle factors in the development of chronic diseases, particularly in the context of cardiovascular health (56).

As technology continues to advance and costs decrease, metabolomics-driven dynamic health management is poised to become a cornerstone of precision public health and personalized health maintenance. The increasing accessibility of metabolic profiling technologies, such as mass spectrometry and nuclear magnetic resonance spectroscopy, enables broader implementation of these strategies in clinical practice. This democratization of metabolic monitoring allows for a more extensive population reach, facilitating the identification of at-risk individuals across diverse demographics. Furthermore, the integration of artificial intelligence and machine learning in analyzing metabolic data can enhance the precision of health interventions, tailoring them to individual metabolic profiles and lifestyle factors. This personalized approach not only optimizes health outcomes but also fosters a more engaged and informed patient population, capable of making decisions that positively impact their health trajectories (57).

In addition, the potential for dynamic metabolic navigation to inform public health initiatives is significant. By utilizing aggregated metabolic data from diverse populations, public health officials can identify trends and risk factors that contribute to the burden of cardiovascular diseases. This information can guide the development of targeted health promotion campaigns and policies that address specific community needs, ultimately improving population health outcomes. As societies increasingly prioritize preventive health strategies, the role of metabolomics in shaping public health initiatives will become increasingly vital, supporting the transition from a reactive to a proactive healthcare system. The promise of metabolomics in enhancing health promotion efforts underscores the importance of interdisciplinary collaboration among healthcare providers, researchers, and policymakers to realize the full potential of this innovative approach (30, 53).

To promote clinical validation of the dynamic navigation paradigm, future research should prioritize three directions: (1) launch large-scale, multicenter, randomized controlled trials in cardiac rehabilitation cohorts to compare the efficacy of metabolomics-guided dynamic intervention with standard care; (2) establish unified metabolomic detection protocols and clinically meaningful metabolite threshold standards; (3) develop low-cost, portable point-of-care testing devices to realize real-time metabolic monitoring at the bedside (58). Only through systematic clinical validation can the dynamic navigation framework be translated into a sustainable and widely applicable precision cardiac rehabilitation model.

## 8 Conclusion

Integrating blood metabolomics into cardiac rehabilitation (CR) marks a transformative shift from standardized protocols to a precise, “ntegrating blood me paradigm”. By leveraging the metabolic fingerprint as a real-time biological navigator, clinicians can address inherent patient heterogeneity, enabling superior risk stratification and objective efficacy evaluation. This approach recognizes CR as an evolving process necessitating continuous adaptation based on fluctuating metabolic states (59).

Despite its potential, significant hurdles remain. Successful clinical translation requires the seamless integration of high-throughput analytical platforms, multi-omics data, and AI-driven algorithms, all of which demand rigorous validation for reliability. Furthermore, addressing cost-effectiveness and fostering interdisciplinary expertise across cardiology, bioinformatics, and digital health are essential for widespread implementation.

Looking ahead, the future of CR lies in an intelligent closed-loop ecosystem. By fusing multi-omics with wearable biosensors and digital therapeutics, this framework facilitates real-time intervention optimization tailored to individual biological signals. Such an adaptive, data-driven model aligns with the broader goals of precision medicine and patient-centered care. Ultimately, blood metabolomics provides the scientific foundation to redefine CR, heralding a new era of individualized management that significantly improves patient outcomes and cardiovascular health milestones.
