# Correction: Dynamic navigation-based precision cardiac rehabilitation via blood metabolomics: from risk stratification to real-time intervention optimization

**DOI:** 10.3389/fcvm.2026.1977500

**Published:** 2026-09-14

**Authors:** Hejuan Hua, Yanbing Chen, Tianyi Yu

**Affiliations:** 1 Department of Cardiology, First People's Hospital of Xiaoshan District, Hangzhou, Zhejiang, China; 2 School of Clinical Medicine, Hangzhou Medical College, Hangzhou, Zhejiang, China; 3 Department of Rehabilitation, First People's Hospital of Xiaoshan District, Hangzhou, Zhejiang, China

**Keywords:** biomarkers, dynamic navigation, intervention optimization, metabolic fingerprint, metabolomics, precision cardiac rehabilitation, risk stratification

Affiliation Department of Cardiology, First people's Hospital of Xiaoshan District, Hangzhou, Zhejiang, China was omitted from the paper. This affiliation has now been added for author Hejuan Hua. Affiliation Department of Rehabilitation, First People's Hospital of Xiaoshan District, Hangzhou, Zhejiang, China was erroneously assigned to author Hejuan Hua. This affiliation has now been removed for author Hejuan Hua. The original version of this article has been updated.

